# Switch to dolutegravir is well tolerated in Thais with HIV infection

**DOI:** 10.1002/jia2.25324

**Published:** 2019-07-11

**Authors:** Orlanda Q Goh, Donn J Colby, Suteeraporn Pinyakorn, Carlo Sacdalan, Eugène Kroon, Phillip Chan, Nitiya Chomchey, Ratchapong Kanaprach, Peeriya Prueksakaew, Duanghathai Suttichom, Rapee Trichavaroj, Serena Spudich, Merlin L Robb, Praphan Phanuphak, Nittaya Phanuphak, Jintanat Ananworanich, on behalf of RV254/SEARCH 010 Study Group

**Affiliations:** ^1^ SEARCH The Thai Red Cross AIDS Research Centre Bangkok Thailand; ^2^ Johns Hopkins Bloomberg School of Public Health Baltimore MD USA; ^3^ Duke‐National University of Singapore Medical School Singapore Singapore; ^4^ The Henry M. Jackson Foundation for the Advancement of Military Medicine Bethesda MD USA; ^5^ United States Military HIV Research Program Walter Reed Army Institute of Research Silver Spring MD USA; ^6^ Department of Retrovirology Armed Forces Research Institute of Medical Sciences United States Component Bangkok Thailand; ^7^ Department of Neurology Yale University New Haven CT USA; ^8^ Department of Global Health University of Amsterdam Amsterdam The Netherlands

**Keywords:** HIV, Asian, dolutegravir, adverse effects, toxicity, hepatitis C

## Abstract

**Introduction:**

Dolutegravir (DTG) is recommended as part of first‐line antiretroviral therapy (ART) for people living with HIV(PLHIV). We sought to determine the rate of adverse events (AEs) and discontinuations among Thais treated during acute HIV infection (AHI) and switched to DTG‐based regimens.

**Methods:**

Thai participants in the SEARCH010/RV254 cohort who initiated ART during AHI and switched to DTG for at least 48 weeks were prospectively observed and included in the analysis. Rates and characteristics of DTG‐related AEs and discontinuations were described.

**Results:**

A total of 313 Thai participants were included in the analysis. The median age was 29 years, 96% were male, 64% had a Bachelor's degree or higher and 16% had a body mass index (BMI) <18.5 kg/m^2^. Participants were on ART for a median of 124 weeks before switching to DTG. The median (IQR) body weight increased from 63 (56 to 70) kg before to 65 (58 to 73) kg (*p* < 0.0001) after 48 weeks of DTG. Forty‐nine (16%) developed DTG‐related AEs, corresponding to an incidence of 16.6 per 100 person‐years. Neuropsychiatric symptoms were most frequently encountered (n = 25, 8%), followed by laboratory abnormalities (n = 16, 5%). Six (2%) discontinued DTG, corresponding to an incidence of 2.4 per 100 person‐years. All discontinuations were due to increased liver enzymes in the presence of hepatitis C virus coinfection. In the multivariate analysis, incident hepatitis C virus infection was the only risk factor for discontinuing DTG (hazard ratio 59.4, 95% CI 8.5 to 297.9, *p* < 0.0001). Neither low BMI nor concurrent abacavir therapy was associated with discontinuation.

**Conclusions:**

DTG was well tolerated with few discontinuations in this cohort of young men. Incident hepatitis C virus infection was a driver of liver‐related AEs leading to discontinuations. In populations at risk, regular testing for hepatitis C virus during ART is recommended to anticipate possible AEs, guide management and improve safety.

## 1 Introduction

Dolutegravir (DTG) is a potent second generation HIV integrase strand transfer inhibitor (INSTI) with a favourable profile of efficacy, safety and tolerability in adults and adolescents 1, 2. It has a high barrier to resistance and low drug–drug interaction potential 3. In light of these factors and its ease of use, DTG is included in all high‐income country guidelines as an initial regimen for people living with HIV (PLHIV) 4, 5.

Generic DTG has been introduced into low‐ and middle‐income countries (LMIC), making it an affordable first‐line option or an alternative therapy in case of efavirenz (EFV) intolerance or resistance in countries where generic DTG is available 6. Low‐cost DTG enables LMIC to simplify treatment regimens, decrease the incidence of side effects and reduce the risk of antiretroviral drug resistance 6, 7. Low‐cost, generic fixed dose combination (FDC) tablets of DTG co‐formulated with tenofovir disoproxil fumarate and lamivudine are now available as the first single daily pill alternative to EFV‐based antiretroviral therapy (ART) 6. Recently, the World Health Organization included DTG as a preferred regimen for initial treatment of HIV infection 8.

Post‐marketing surveillance has raised concern over DTG‐related adverse events (AEs) in PLHIV in high‐income countries. Neuropsychiatric AEs including headache, dizziness, sleep disturbances, mood disturbances, suicidal ideation and cognitive problems were reported. Unintentional weight gain was also described 9. Recently, an observational study in Botswana showed a higher rate of neural tube defects among infants born to women taking DTG during conception at 0.9% versus 0.1% in those taking other antiretroviral drugs 10.

In US, European and Australian cohorts, AEs have led to DTG discontinuation in 1.6% to 13.7% of people 3, 11, 12, 13, 14, 15, 16. This is higher than a previously reported EFV discontinuation rate of 4.3% in a Thai ART‐naïve cohort 17. Limited published data on switching to DTG‐based regimens also suggest significant rates of AEs and discontinuations 18, 19. To the best of our knowledge, there is no published report of safety and tolerability of DTG in Asians. People with lower body weight may be at risk for AEs from antiretrovirals 20, 21, 22. Such AEs may also be compounded by comorbidities 23. Here, we sought to determine the rate of DTG AEs and discontinuations in a cohort of predominantly young Thai males who are healthy and have low body weight. They were treated with ART since acute HIV infection (AHI) and subsequently switched to DTG‐based regimens.

## 2 Methods

### 2.1 Participants

Participants were enrolled in the prospective SEARCH010/RV254 AHI cohort study in Bangkok, Thailand 24 (clinicaltrials.gov NCT00796146) since 2009. Immediate ART was offered under a separate protocol (NCT00796263). The initial preferred ART regimen was efavirenz (EFV), tenofovir (TDF) and either emtricitabine (FTC) or lamivudine (3TC), which was available as a single tablet daily regimen. Substitutions of antiretroviral drugs occurred as clinically indicated for intolerance or resistance. In March 2017, the cohort's preferred ART regimen changed to DTG, 3TC and abacavir (ABC). The regimen was taken as two pills once per day as FDC ABC/3TC/DTG was not yet available in Thailand. All participants were pre‐screened for HLA B*5701. TDF was substituted for ABC in participants positive for HLA B*5701 or hepatitis B surface antigen (HBsAg). Participants had the options to access free HIV treatment in the public sector or obtain alternative ART in the study if clinically indicated.

This analysis included SEARCH010/RV254 participants who switched to a DTG‐based regimen and had at least 48 weeks of follow‐up after the switch. Data used for analysis were censored on 30 April 2018. All participants provided written informed consent. The study was approved by the institutional review boards of Chulalongkorn University, Bangkok, Thailand, and the Walter Reed Army Institute of Research, MD, USA.

### 2.2 Clinical and laboratory characterisation

Laboratory testing was performed at baseline and during follow‐up (at least every 12 weeks for HIV RNA and CD4 count, every 24 weeks for liver and renal function, and every 48 weeks for hepatitis B and C serology). Infection with hepatitis B virus (HBV) or hepatitis C virus (HCV) was confirmed with plasma viral load testing.

Relevant treatment history included pre‐DTG ART regimen and history of previous medication switches. Illicit drug use was defined as any prior recreational use by any route of glue or petrol, cannabis derivatives, cocaine derivatives, methamphetamine derivatives, khat, heroin, lysergic acid diethylamide (LSD) or amyl nitrate derivatives (poppers).

All AEs were graded following the Division of AIDS (DAIDS) Table for Grading the Severity of Adult and Pediatric Adverse Events, Version 2.1 25. DTG was discontinued if any of these protocol‐defined criteria as per the manufacturer were reached: alanine aminotransferase level (ALT) >8 times the upper limit of normal (ULN), ALT >5 times but <8 times the ULN for >2 weeks (or if the participant could not be monitored weekly), ALT >3 times the ULN and bilirubin >2 times the ULN or ALT >3 times the ULN with symptoms of acute hepatitis or hypersensitivity. DTG discontinuation was also considered if participants had intolerable clinical side effects, virological resistance, or a preference for an alternative regimen. Participants were considered to have an AE related to DTG if a study physician deemed it as being possibly, probably or certainly due to DTG. All discontinuations during this observation period were included in the analysis Table 1.

**Table 1:** Characteristics of participants

	At AHI	At time of DTG switch	48 Weeks after switch
Age (years), median (IQR)	26 (23 to 31)	29 (25 to 35)	30 (26 to 36)
Male, n (%)	301 (96.2)	‐	‐
MSM, n (%)	265 (84.7)	‐	‐
Bachelor's degree or higher, n (%)	201 (64.2)	‐	‐
HIV RNA‐1 (Log_10_ copies/mL), median (IQR)	5.9 (5.3 to 6.8)	‐	‐
HIV RNA‐1 <50 (copies/mL), n (%)	‐	301 (96.2)	306 (97.8)^a^
CD4 cell count (cells/mm^3^), median (IQR)	371 (267 to 495)	658 (539 to 817)	692 (551 to 885)^b^
Time on ART (in weeks), median (IQR)	‐	124 (62 to 194)	172 (110 to 242)
Time on DTG (in weeks), median (IQR)	‐	‐	57 (54 to 60)
Concurrent ABC/3TC with DTG, n (%)	‐	0 (0.0)	264 (84.3)

IQR, Interquartile range.

^a^ *p* = 0.3018 comparing before and after DTG switch; ^b^ *p* = 0.0002 comparing before and after DTG switch.

### 2.3 Analyses

Incidence of DTG‐related AEs and the discontinuation of DTG were calculated using the number of events divided by the cumulative years of follow‐up. Participants were censored at the time of the onset of events or 30^th^ April 2018, whichever occurred first. Time to AEs related to DTG and time to DTG discontinuation were described using Kaplan‐Meier functions. Differences in the survival functions between groups were assessed by the log rank tests. Cox proportional hazards model was used to determine factors associated with the incident of AEs or DTG discontinuation. The candidate factors include age at DTG switch, sex, recent illicit drug use, bachelor's degree or higher, low BMI at time of switch, a high pre‐switch ALT level, HBV and HCV status, incident HCV infection, prior ART for >96 weeks, prior medication switches, concurrent therapy with ABC and more than 57 weeks on DTG (median time on DTG in the cohort). The differences before and after DTG switch were compared using the Wilcoxon signed‐rank test. Proportion of participants with viral load <50 copies per mL before and after switch were compared using McNemar's exact tests. All tests were two‐sided with significance level of 0.05. Statistical analyses were performed using StataCorp 2017 (*Stata Statistical Software: Release 15*. College Station, TX: StataCorp LLC). Figures were created using GraphPad Prism version 7.0 for Windows (GraphPad Software, La Jolla California USA).

## 3 Results

### 3.1 Study population, weight changes and viral suppression

At the time of analysis there were 411 participants in the SEARCH010/RV254 cohort with at least 48 weeks of ART experience. Of these, 78 had less than 48 weeks follow‐up on DTG, 9 were of non‐Thai ethnicity and 11 had not switched to DTG. Reasons for not switching to DTG were participant preference to not switch to a new regimen with a higher pill burden (n = 5), and elevated ALT due to hepatitis C (n = 4), hepatitis A (n = 1) or undetermined aetiology (n = 1).

There were a total of 313 Thai participants who switched to a DTG‐based regimen and had at least 48 weeks of follow‐up after the switch. At AHI, the median age was 26 years and 96% were male. Median HIV RNA was 5.9 log_10_copies/mL and CD4 was 371 cells/mm^3^. Fifty‐three percent were in Fiebig stage I/II, 43% in Fiebig stage III/IV and 4% in Fiebig stage V.

At the time of switch to DTG, participants had been on ART for a median of 124 weeks. The majority (n = 254, 81%) switched from an EFV‐based regimen with 30 (10%), 16 (5%) and 13 (4%) switching from lopinavir/ritonavir, rilpivarine and other regimens respectively. One‐quarter (27%) had a history of previous ART substitution. HLA‐B*5701 was positive in three participants (1%).

By 30 April 2018, participants had been on DTG for a median of 57 weeks. The majority (n = 264, 84%) received ABC/3TC as the nucleoside reverse transcriptase inhibitor (NRTI) backbone. Illicit drug use was reported by 17% in the previous year and 31% over lifetime. By 48 weeks, 98% had HIV RNA <50 copies/mL, compared with 96% prior to switching (*p* = 0.3018). The median CD4 was higher 48 weeks after switch at 692 cells/mm^3^ compared with 658 cells/mm^3^ prior (*p* = 0.0002).

Prior to switching, the prevalence of HBV infection was 7%, and HCV infection was 2%. The period after switch to DTG coincided with a dramatic increase in HCV infection in the cohort, with 13 (4%) incident cases of HCV. There was no incident case of HBV infection.

**Figure 1:**
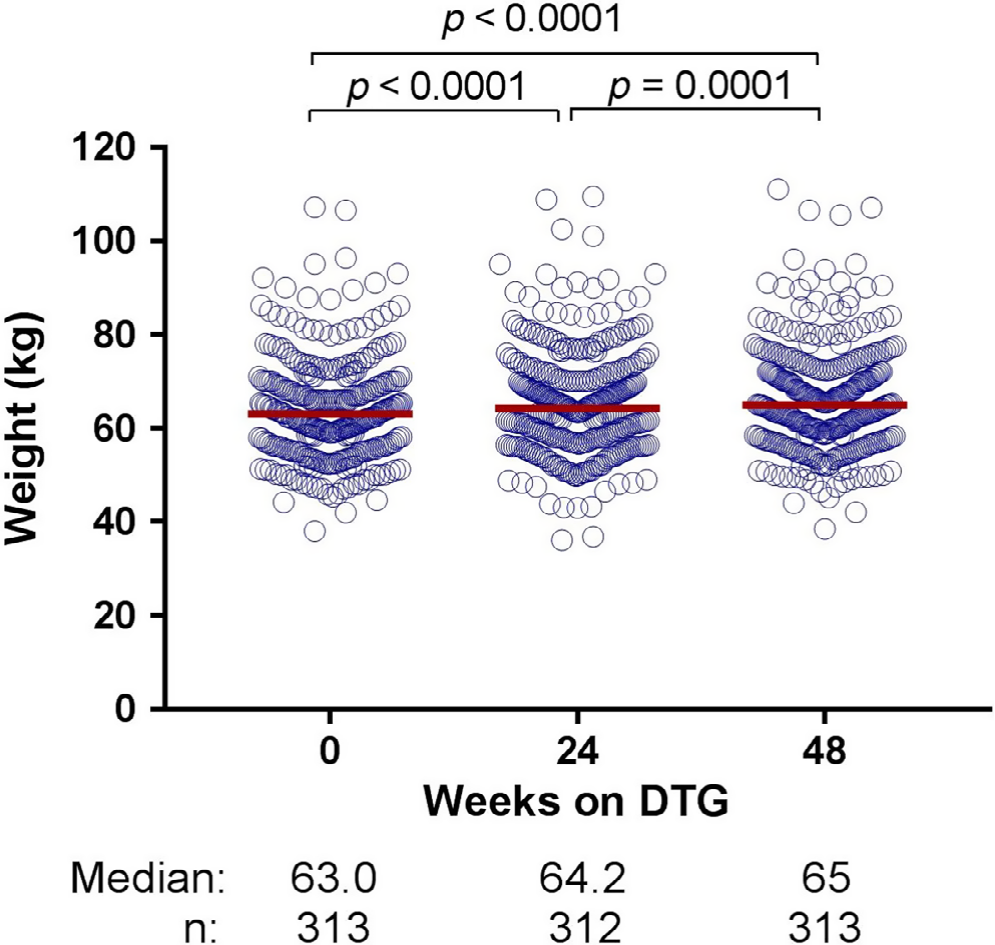
Body weight at baseline and after dolutegravir‐based regimen

Although the median (interquartile range, IQR) BMI of 21.4 (19.3 to 23.6) kg/m^2^ was within the normal range for Asians, 16% of the cohort was underweight with a BMI <18.5 kg/m^2^ at DTG switch. The median (IQR) BMI increased to 22.0 (20.1 to 24.5) kg/m^2^ after 48 weeks of DTG (*p* < 0.0001). The median (IQR) body weight increased from 63 (56 to 70) kg before to 65 (58 to 73) kg (*p* < 0.0001) after 48 weeks of DTG (Figure 1). This weight increased by a mean (standard deviation, SD) of 1.6 (±3.4) kg or 3 (±5)% after 24 weeks (*p* < 0.0001), and by a mean (SD) of 2.3 (±3.8) kg or 4 (±6)% by 48 weeks after DTG switch (*p* <0.0001).

### 3.2 Safety and tolerability of DTG

**Figure 2:**
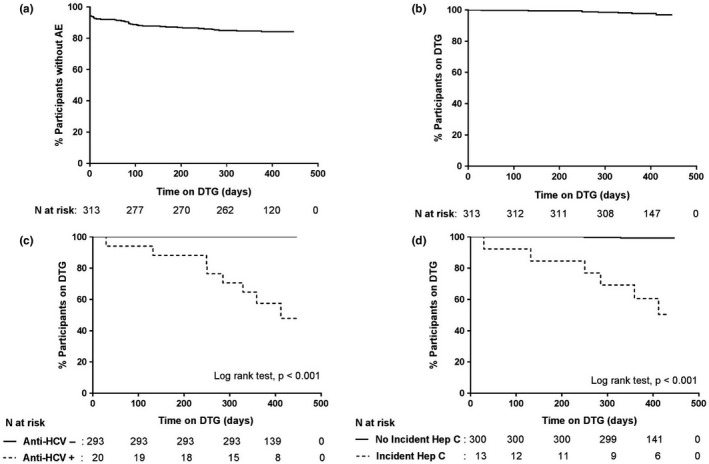
Proportions of participants on dolutegravir (**a**) without adverse events (**b**) without dolutegravir discontinuation, (**c**) positive or negative anti‐HCV, (**d**) with or without incident Hep C.

By 48 weeks, 49 (16%) participants had developed AEs related to DTG, corresponding to an incidence (95% confidence interval, CI) of 16.6 (12.5 to 22.0) AEs per 100 person‐years (Figure 2A).

In total, there were 52 unique AEs observed among 49 participants. They were classified according to the DAIDS criteria in Table 2 25. Overall, 81% (n = 42) of AEs were mild in severity (grade 1). Only 6% (n = 3) of AEs were severe including one grade 3 low‐density lipoprotein (LDL), one grade 3 creatinine clearance and one grade 4 alanine transaminase (ALT). One‐third (37%) of AEs occurred within one day and 50% occurred within 24 (IQR 1 to 106) days of initiating DTG.

**Table 2:** List of AEs related to DTG

AE related to DTG	Participants, n (%)	Grade, n (%)				Discontinuation, n (%)
		1	2	3	4	
Neuropsychiatric	25 (8.0)	22 (7.0)	3 (1.0)	0 (0.0)	0 (0.0)	0 (0.0)
Headache	3 (1.0)	3 (1.0)	0 (0.0)	0 (0.0)	0 (0.0)	0 (0.0)
Dizziness	11 (3.5)	10 (3.2)	1 (0.3)	0 (0.0)	0 (0.0)	0 (0.0)
Insomnia	10 (3.2)	9 (2.9)	1 (0.3)	0 (0.0)	0 (0.0)	0 (0.0)
Bipolar affective disorder	1 (0.3)	0 (0.0)	1 (0.3)	0 (0.0)	0 (0.0)	0 (0.0)
Gastrointestinal	5 (1.6)	5 (1.6)	0 (0.0)	0 (0.0)	0 (0.0)	0 (0.0)
Nausea	3 (1.0)	3 (1.0)	0 (0.0)	0 (0.0)	0 (0.0)	0 (0.0)
Vomiting	1 (0.3)	1 (0.3)	0 (0.0)	0 (0.0)	0 (0.0)	0 (0.0)
Dyspepsia	1 (0.3)	1 (0.3)	0 (0.0)	0 (0.0)	0 (0.0)	0 (0.0)
Dermatologic	2 (0.6)	2 (0.6)	0 (0.0)	0 (0.0)	0 (0.0)	0 (0.0)
Rash	1 (0.3)	1 (0.3)	0 (0.0)	0 (0.0)	0 (0.0)	0 (0.0)
Dry skin	1 (0.3)	1 (0.3)	0 (0.0)	0 (0.0)	0 (0.0)	0 (0.0)
Systemic and others	4 (1.3)	3 (1.0)	1 (0.3)	0 (0.0)	0 (0.0)	0 (0.0)
Fatigue	1 (0.3)	0 (0.0)	1 (0.3)	0 (0.0)	0 (0.0)	0 (0.0)
Hot flushes	2 (0.6)	2 (0.6)	0 (0.0)	0 (0.0)	0 (0.0)	0 (0.0)
Palpitations	1 (0.3)	1 (0.3)	0 (0.0)	0 (0.0)	0 (0.0)	0 (0.0)
Laboratory	16 (5.1)	10 (3.2)	3 (1.0)	2 (0.6)	1 (0.3)	1 (0.3)
High ALT	14 (4.5)	10 (3.2)	3 (1.0)	0 (0.0)	1 (0.3)	1 (0.3)
Low creatinine clearance	1 (0.3)	0 (0.0)	0 (0.0)	1 (0.3)	0 (0.0)	0 (0.0)
High LDL	1 (0.3)	0 (0.0)	0 (0.0)	1 (0.3)	0 (0.0)	0 (0.0)
Total number of AEs	52 (100.0)					

Neuropsychiatric AEs were most commonly encountered (8%). The most frequently reported symptoms were dizziness (4%) and insomnia (3%). No one reported suicidal ideation. One participant had existing symptoms of bipolar affective disorder that was diagnosed while on DTG and was controlled with oral quetiapine. The second most common DTG‐related AEs were laboratory abnormalities (5%). Raised ALT was the main abnormality (4.5%).

A total of eight participants (3%) discontinued DTG. Six discontinuations (2%) occurred within 48 weeks of DTG initiation and the other two occurred shortly thereafter (weeks 50 and 51). The median time to DTG discontinuation was 268 days (IQR 191 to 344). The incidence of DTG discontinuation (95% CI) was 2.4 (1.2 to 4.7) per 100 person‐years (Figure 2B).

All eight discontinuations were due to protocol‐defined criteria for increased ALT. All occurred in the context of HCV coinfection. Six had incident HCV infections (five cases of isolated incident HCV infection and one with simultaneous incident HCV and hepatitis A virus infection) while on DTG. Their median (IQR) HCV RNA level at diagnosis was 6.5 (5.6 to 7.0) log_10_ IU/mL. Median (range) peak ALT was 665 (513 to 1003) U/L. None of the six had clinical symptoms of hepatitis and in all the ALT improved after DTG discontinuation.

Two participants had existing HCV/HIV coinfection prior to DTG switch. One had a recent HCV infection with HCV RNA level of 6.1 log_10_ IU/mL two months prior to DTG switch. His HCV RNA level was 6.59 log_10_ IU/mL after the DTG switch and just prior to discovery of ALT level of 419 U/L on routine testing at 47 weeks following DTG. This participant had no clinical symptoms of hepatitis. The other participant likely had an indolent HCV coinfection with repeated prior HCV RNA levels <15 IU/mL but developed clinical hepatitis 35 weeks following DTG with an ALT level of 854 U/L and HCV RNA of 380 IU/mL. His symptoms resolved, ALT normalized and HCV RNA became undetectable following DTG discontinuation without specific HCV treatment. The study physician deemed his raised ALT level as possibly due to DTG.

The incidence rate of DTG discontinuation was 0 per 100 person‐years for HCV seronegative and 42.3 per 100 person‐years for HCV seropositive participants (*p* < 0.0001, log rank test) (Figure 2C). When analysed by incident HCV infection, the incidence rate of discontinuation was 0.6 for those without incident infection, and 50.7 per 100 person‐years for those with incident infection (*p* < 0.0001, log rank test) (Figure 2D).

### 3.3 Factors associated with DTG‐related AEs and DTG discontinuations

There was no statistically significant difference between those with and without DTG AEs for age, sex, sexual preferences, recent illicit drug use, education level, BMI, HBV or HCV status, pre‐ART or pre‐switch HIV RNA‐1 level or CD4 cell count, time on ART, prior regimens, history of previous medication switches, time on DTG or concurrent therapy with ABC/3TC. In the univariate and multivariate Cox regression models, there was no association between any of the above covariates and higher incidence of AEs, nor was there an association with neuropsychiatric AEs in a subgroup analysis.

In the univariate analysis for the discontinuation of DTG, associations were observed for incident HCV infection (*p* < 0.001) and history of medication switches (*p* = 0.008) (Table 3). In the multivariate analysis, only incident HCV infection was associated with DTG discontinuation with a hazard ratio (95% CI) of 58.0 (11.3 to 299.2) (*p* < 0.0001) (Table 3).

**Table 3:** Cox regression for discontinuation of DTG

Covariate	Univariate HR (CI)	*p* value	Multivariate HR (CI)	*p* value
Age (years old) at switch ≥40	1.10 (0.13 to 8.92)	0.090	‐	‐
Illicit drug use within one year of DTG	1.67 (0.34 to 8.27)	0.531	‐	‐
Bachelor's degree or higher	0.88 (0.21 to 3.70)	0.864	‐	‐
BMI ≤18.5kg/m^2^	1.77 (0.36 to 8.79)	0.483	‐	‐
Pre‐switch ALT >55 U/L	1.95 (0.39 to 9.67)	0.414	‐	‐
Seropositive for HBsAg	1.99 (0.24 to 16.22)	0.519	‐	‐
Incident HCV infection	81.56 (16.39 to 405.92)	<0.001	58.01 (11.25 to 299.20)	<0.001
ART >96 weeks	1.06 (0.25 to 4.43)	0.937	‐	‐
Previous history of medication switches	8.72 (1.75 to 43.26)	0.008	4.51 (0.88 to 23.16)	0.071
Concurrent ABC with DTG	0.55 (0.11 to 2.71)	0.460	‐	‐
≥57 weeks on DTG	1.93 (0.37 to 9.94)	0.433		

CI, confidence interval; HR, hazard ratio.

## 4 Discussion

DTG was well tolerated in this cohort of acutely treated young Thai males after switching from a prior ART regimen. Approximately one in six people (16%) had an AE and most were mild in severity. Neuropsychological symptoms were most common. Discontinuation rate was low at 2%, and all were due to elevated ALT among people with HCV‐coinfection.

The rate of DTG‐related AEs is comparable to those reported in previous switch studies. Drug‐related AEs ranged between 13 and 22% in previous switch trials 17, 18, 25. In the SWORD‐1 and SWORD‐2 studies that randomized virally suppressed participants to DTG‐rilpivirine or continuing on current ART, 19% of the former group reported drug‐related AEs compared to 2% in the latter group 18. Another randomized trial observed AEs in 12.8% of people who switched to DTG versus 7.2% in those who maintained a protease inhibitor regimen 26.

The DTG discontinuation rate in our cohort is lower than the above‐mentioned studies. In these studies, 3% to 4% of participants had AEs that led to DTG discontinuation by week 48. One observational study among mostly males in Australia who switched to DTG regimen for simplification observed a cumulative DTG discontinuation rate of 3.2% at 12 months 16.

Noticeably, the DTG discontinuation rates were higher at 11% to 15% among cohorts with ART‐naive individuals 12, 14, 15. In these studies, 4% to 14% (median = 9%) of participants had AEs that resulted in DTG discontinuation 3, 12, 14, 15.

The superior tolerability to DTG among ART‐experienced people could be related to the absence of neuropsychological symptoms that often accompany a new diagnosis of HIV 27, an already induced liver metabolism or a higher tolerance of AEs. Our cohort had had more than two years on ART and a decrease of clinically relevant depressive and anxiety symptoms with time, concurrent with ART 28. A fair number (27%) had a prior drug switch due to intolerance or resistance (mostly switching from EFV to alternative agents).

This low DTG discontinuation rate despite a low tolerance for liver enzyme elevation, also suggests that it is better tolerated than EFV in the Thai population. Although there are no data on the tolerability of switching to EFV in Thailand, our group has shown a 4% EFV discontinuation rate at 24 weeks in ART‐naïve Thai adults 17.

Our study differs from earlier studies that have suggested that female sex, older age, low body weight and concurrent therapy with ABC were risk factors for discontinuation of DTG due to AEs 3, 12, 14, 15. Our discontinuation rate was low despite 16% being underweight (BMI < 18.5kg/m^2^) at DTG switch. Neither low BMI nor concurrent therapy with ABC was associated with discontinuations by multivariable analysis. Instead, incident HCV infection was the only significant risk factor for DTG discontinuation.

Although only 6% of our participants were seropositive for HCV by the end of the study, 65% of these were incident cases that occurred during the analysis period. Between 2014 and 2017, the incidence of HCV in our cohort rose from 0.9 to 3.4 per 100‐person years (unpublished data). This is consistent with increasing incidences of HCV infection described among MSM with HIV on multiple continents 29, 30, and with reports of increased drug use, especially crystal methamphetamine, among MSM in Thailand 31.

Although we could not definitively rule out DILI as a factor for elevated ALT in participants who discontinued DTG, we believe that in the majority of cases the cause of the abnormal liver enzymes was incident HCV infection that coincidentally occurred after the DTG switch. Evidence for this includes the temporal association between incident HCV infection and rising ALT in six of the eight participants discontinuing DTG. Moreover, the multivariate analysis showed the only significant factor to be incident HCV with a high hazard ratio of 58. Furthermore, none of the 293 participants without HCV infection met criteria for stopping DTG.

Two participants who discontinued DTG had HCV infection prior to the medication switch: in these cases, the relative contributions of HCV infection and DTG switch to the abnormal liver function could not be determined.

We therefore recommend screening for HCV at least annually in populations with known demographic risk factors for prevalent and incident HCV. There should be a lower clinical threshold for more frequent screening especially when a new ART drug is being prescribed, and increased caution for initiating a potentially hepatotoxic drug in patients with pre‐existing HCV infection.

As expected, neuropsychiatric symptoms were the most common AEs to DTG (8% of participants and 48% of AEs). Contrary to existing literature, none resulted in DTG discontinuation. In pooled data from five randomized clinical trials in the OPERA trial, four of which were in ART‐naïve cohorts, 19% reported psychiatric AEs 11. In the SWORD trial that randomized ART experienced participants to DTG/RPV versus maintenance of current ART, 1% discontinued DTG due to neuropsychiatric AEs 19. A cohort study from Switzerland reported about 2% discontinuation of DTG due to neuropsychiatric AEs in ART‐naïve individuals. Retrospective observational studies including both ART‐naïve and experienced people have reported 6% to 14% DTG discontinuations due to neuropsychiatric symptoms 3, 12, 15. The unexpectedly low discontinuation rate due to neuropsychiatric AEs could be explained by either a truly lower incidence of symptoms, or by a higher tolerance of symptoms in our cohort members.

The mean weight gain of 2.3kg (3.7%) over 48 weeks was noticed by participants and was tolerated by all. A recent study reported a weight gain of 5.3kg in 18 months among participants who were virologically suppressed and mostly Caucasian and who switched from an EFV‐based regimen to DTG 9. However, this was not observed in other DTG trials of ART‐naïve 23, 32, 33 or–experienced participants 34. Weight gain could be from a change in body fat distribution and metabolism 9 or insulin sensitivity 35. Further research is needed to confirm the impact of DTG and INSTI drugs on weight gain and possibly related metabolic comorbidities.

This analysis has certain limitations. Our findings may not be applicable to populations other than young MSM. We are unable to perform robust analysis to confirm or refute the role of sex or age on DTG AEs and discontinuation. The effect of HCV prevalent infection on DTG tolerability may have been different if HCV was treated. All of our participants were treated for HIV early during acute HIV infection and other comorbidities were rare. Hence, pertinent questions on the safety of DTG for key populations in LMIC 7 such as women of child‐bearing age, HIV‐tuberculosis coinfection and those with severe immunodeficiency remain unanswered.

To increase the safety of ART in PLHIV, we recommend regular screening of HCV serology in HCV endemic regions and in sub‐populations at high risk for incident hepatitis C infection.

## 5 Conclusions

This prospective study in a Thai population shows that DTG is as well or better tolerated than has been reported in Western cohorts.

By focusing on people who were switched from other ART regimens and including participants who were coinfected with HBV and HCV, our study addresses a relevant clinical question on the suitability of DTG as part of a simplification or switch regimen where viral hepatitis is prevalent. The low AE and discontinuation rate in our population in which one in four had prior drug switches supports the potential for DTG to improve treatment satisfaction, facilitate treatment adherence and improve therapeutic outcomes 19.

## Competing interests

JA has received honoraria for participating in advisory meetings for ViiV Healthcare, Gilead, Merck, Roche and AbbVie. SS reports grants from NIH/NIMH/NINDS and directs a clinical research study unrelated to these projects through the AIDS Clinical Trials Group that receives study medication from ViiV Healthcare, Inc. Other authors declare no conflict of interest.

## Authors’ contributions

OG, DC and JA conceived, designed and drafted the article. OG, DC, CS, EK, PC, JI, RT, TL, NC and NP participated in the study design, coordination, laboratory testing, data interpretation and clinical care of the participants. OG, DC and SP compiled and analysed the data. All authors read, provided input in the article and approved the final article.

## Appendix 1

### 1.1

The RV254/SEARCH 010 Study Group includes the following team members from SEARCH/TRC‐ARC/HIV‐NAT: Nipat Teeratakulpisarn, Supanit Pattanachaiwit, Mark de Souza, James Fletcher, Ponpen Tantivitayakul, Kultida Poltavee, Jintana Intasan, Tassanee Luekasemsuk, Hathairat Savadsuk, Somporn Tipsuk, Suwanna Puttamsawin, Khunthalee Benjapornpong, Nisakorn Ratnaratorn, Kamonkan Tangnaree, Chutharat Munkong, Rommanus Thaimanee, Patcharin Eamyoung, Sasiwimol Ubolyam; from AFRIMS: Robert O'Connell, Alexandra Schuetz, Denise Hsu, Tanyaporn Wansom, Siriwat Akapirat, Bessara Nuntapinit, Nantana Tantibul, Nampueng Churikanont, Saowanit Getchalarat; MHRP: Nelson Michael, Trevor Crowell, Ellen Turk, Corinne McCullough, Oratai Butterworth, Madelaine Ouelette, Mark Milazzo, Leigh Anne Eller.
